# Supplementary material for: Regional Difference in the Association between the Trajectory of Selenium Intake and Hypertension: A 20-Year Cohort Study
Source: Nutrients. 2021 Apr 29;13(5):1501. doi: 10.3390/nu13051501 (PMC8146509; doi:10.3390/nu13051501)
Supplement: Supplementary file 1 [file nutrients-13-01501-s001.zip › nutrients-1175771-supplementary.pdf]

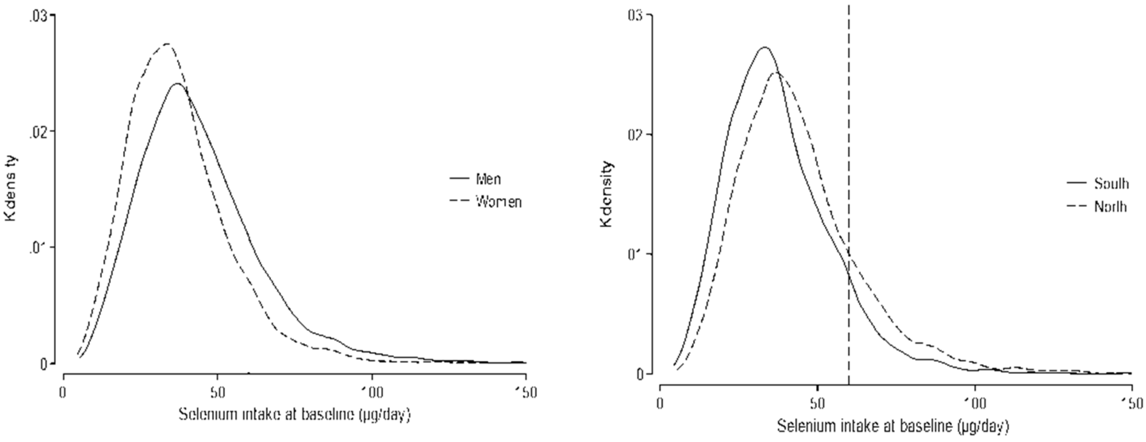

Supplement Figure 1: Distribution of selenium intake at baseline among participants in the China Health and Nutrition Survey

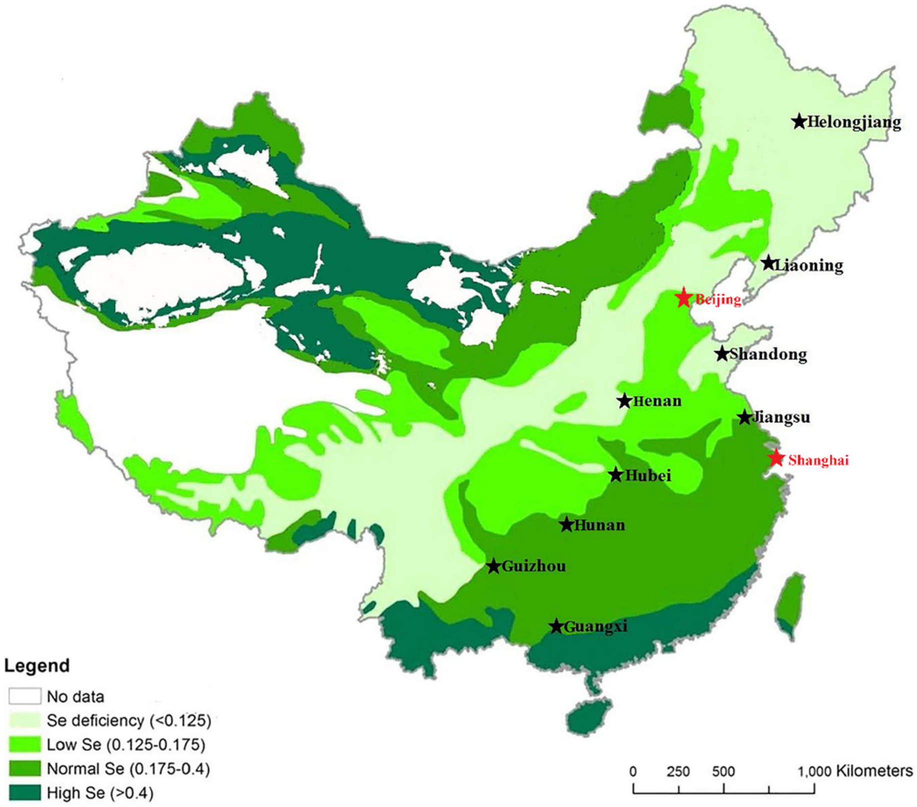

Supplement Figure 2. The position of provinces investigated on the soil Se distribution map of China. Data source: Sun GX, Meharg AA, Li G, Chen Z, Yang L, Chen SC, Zhu YG. Distribution of soil selenium in China is potentially controlled by deposition and volatilization? Sci Rep. 2016 Feb 17;6:20953. doi: 10.1038/srep20953. PMID: 26883576; PMCID: PMC4756323. Compared to the published figure, we removed the legend showing desertification (irrelevant to this study) and added the positioning of nine provinces as well as Beijing and Shanghai. Creative Commons license. http://creativecommons.org/licenses/by/4.0/

Supplement Table 1. Baseline sample characteristics by trajectory of selenium intake: CHNS (*N* = 10,025)

Factor	G1	G2	G3	G4	*p*-Value
N	**7149**	**2364**	**299**	**213**	
Se intake (μg/d), mean (SD)	35.7(18.6)	51.2(21.3)	51.2(22.5)	87.6(34.0)	<0.001
Energy intake (kcal/d), mean (SD)	2434.1(666.2)	2724.7(744.9)	2631.5(681.2)	2998.5(880.1)	<0.001
Fat intake (g/d), mean (SD)	60.9 (35.5)	73.9 (39.5)	79.1 (44.1)	86.8 (43.5)	<0.001
Protein intake (g/d), mean (SD)	69.6 (21.3)	85.1 (25.0)	82.2 (23.6)	102.9 (29.3)	<0.001
Carbohydrate intake (g/d), mean (SD)	397.3 (132.2)	423.6 (159.2)	392.5 (138.4)	438.8 (189.9)	<0.001
Sodium intake (g/d), mean (SD)	6.4 (5.3)	7.1 (5.6)	7.0 (4.8)	8.0 (5.4)	<0.001
Age (years), mean (SD)	40.4 (14.0)	37.2 (11.5)	36.4 (11.6)	38.5 (12.2)	<0.001
BMI (kg/m^2), mean (SD)	21.7 (2.8)	22.3 (2.9)	22.6 (2.8)	22.9 (2.7)	<0.001
Sex					<0.001
Males	42.8%	63.5%	75.6%	77.0%	
Females	57.2%	36.5%	24.4%	23.0%	
Income					<0.001
Low	34.6%	25.3%	19.5%	16.3%	
Medium	33.5%	29.9%	26.9%	26.9%	
High	31.8%	44.8%	53.5%	56.7%	
Education					<0.001
Low	54.9%	37.5%	32.4%	34.2%	
Medium	29.2%	36.9%	33.2%	37.4%	
High	15.9%	25.5%	34.4%	28.3%	
Urbanization					<0.001
Low	50.5%	40.2%	33.4%	26.3%	
Medium	27.8%	29.4%	28.4%	35.7%	
High	21.8%	30.4%	38.1%	38.0%	
Smoking					<0.001
Non smoker	69.1%	56.4%	54.7%	46.8%	
Ex-smokers	1.4%	2.2%	1.8%	1.1%	
Current smokers	29.5%	41.4%	43.5%	52.1%	
Physical activity (MET-hrs/week), mean (SD)	223.6 (188.9)	199.9 (165.1)	174.1 (138.8)	173.6 (114.5)	<0.001
